# Simple size-controlled synthesis of Au nanoparticles and their size-dependent catalytic activity

**DOI:** 10.1038/s41598-018-22976-5

**Published:** 2018-03-15

**Authors:** Petr Suchomel, Libor Kvitek, Robert Prucek, Ales Panacek, Avik Halder, Stefan Vajda, Radek Zboril

**Affiliations:** 1 Department of Physical Chemistry, Regional Centre of Advanced Technologies and Materials, Faculty of Science, Palacky University Olomouc, Slechtitelu 27, 783 71 Olomouc, Czech Republic; 2 Materials Science Division, Argonne National Laboratory, 9600 South Cass Avenue, Lemont, Illinois 60439 USA; 3 Institute for Molecular Engineering, The University of Chicago, 5640 South Ellis Avenue, Chicago, Illinois 60637 USA

## Abstract

The controlled preparation of Au nanoparticles (NPs) in the size range of 6 to 22 nm is explored in this study. The Au NPs were prepared by the reduction of tetrachloroauric acid using maltose in the presence of nonionic surfactant Tween 80 at various concentrations to control the size of the resulting Au NPs. With increasing concentration of Tween 80 a decrease in the size of produced Au NPs was observed, along with a significant decrease in their size distribution. The size-dependent catalytic activity of the synthesized Au NPs was tested in the reduction of 4-nitrophenol with sodium borohydride, resulting in increasing catalytic activity with decreasing size of the prepared nanoparticles. Eley-Rideal catalytic mechanism emerges as the more probable, in contrary to the Langmuir-Hinshelwood mechanism reported for other noble metal nanocatalysts.

## Introduction

Gold nanoparticles (Au NPs) have a wide spectrum of potential applications due to their unique properties, which are in many ways different compared to its bulk form. They find use in laboratories such as basic substrate for bio-sensing and bio-imaging^1–5^, in Surface Enhanced Raman Scattering (SERS) as one of the most effective substrate^6–8^, or in the detection of toxic compounds^9–12^. Although gold in bulk is poorly active as a catalyst, in the form of gold nanoparticles exhibits outstanding catalytic activity^13,14^, especially in redox reactions, such as in the catalytic low-temperatures oxidation of carbon monoxide^15–17^, in oxidation of alcohols^18^, hydrogenation of 4-nitrophenol^19–21^, or in selective epoxidation reactions^22,23^. Au NPs are also often referred as excellent catalyst for reactions based on alkyne activation due to Lewis acid nature of gold^24,25^.

The catalytic activity of Au NPs depends on several factors, and is primarily determined by particle size, i.e. the surface area of nanoparticles which is inversely proportional to the particle size. For example, Tsunoyama and coworkers proved significant increase in the rate of catalytic oxidation of p-hydroxybenzyl alcohol with decreasing Au-PVP (polyvinylpyrrolidone) particle size^26^. Using a variety of organic reactions (silane oxidation, alcohol oxidation and reductive amination), Jawale *et al*. evaluated the differences in catalytic activity between small (3 nm) and larger (20 nm) Au NPs supported on carbon nanotubes. In all cases, the observed reaction rates were at least 5.5 times higher on the smaller Au NPs than on the larger ones^27^. Typical model reaction used for primary evaluation of catalytic activity of the metal nanoparticles is nitroaromatic reduction^28^. Aromal and coworkers have observed a complete reduction of 4-nitrophenol after 30 minutes in the presence of 20 nm Au NPs, while the reaction was accomplished within only 15 minutes under action of 15 nm size Au NPs^29^. Although it is possible to prepare extremely small nanoparticles with diameters of units of nm^30^ or even smaller down to subnanometer size^31^, it has been reported that the catalytic activity of Au NPs does not always monotonically grow with their decreasing particle size. For example, Lin *et al*. has found that the highest catalytic activity in 4-nitrophenol reduction was not observed with the smallest NPs of 1.7 nm size, but larger nanoparticles with 3.4 nm diameter were more active^32^, which in this particular reaction may hint to different (in this case higher) activity of facet atoms with respect to the activity of the edge/corner atoms of the particle. Similar behavior of gold nanoclusters, namely peaking of activity was reported also by Valden *et al*. in CO oxidation by Au NPs^17^, though we need to note recent debates in the open literature about the possible role of sub-nanometer Au clusters in catalytic activity of gold^33,34^ particles not always identified aside the nanosized ones. Corma *et al*. found out, based on theoretical approach confirmed by experimental tests, that geometric and electronic differences between gold clusters comprising a few atoms and gold nanoparticles of 1 nm or larger determine their activity and selectivity. Typically, small planar clusters are highly active for reactions involving activation of the C-C multiple bonds in alkenes and alkynes by means of Lewis acid-base interactions, and on the other hand 3D gold nanoparticles have better catalytic performance in redox reactions involving bond dissociation by oxidative addition and new bond formation by reductive elimination^35^.

According to the mentioned dependence of catalytic activity of different sized Au NPs, it is necessary to find robust and reliable procedure for the size-selective preparation of Au NPs, moreover with narrow size distribution. Au NPs are prepared commonly by the several basic methods, from which Turkevich’s method producing 15 nm sized nanoparticles using citrate as reductant^36^ or Martin’s method producing 3–5 nm sized nanoparticles using NaBH_4_ as reductant^37^ are mostly used in practice. Many other procedures producing single sized spherical Au NPs, e.g. 12 nm^38^, 13 nm^39^ or 21 nm^40^ were published previously, but the most of these methods do not make possible size managed preparation of Au NPs. Brinas *et al*. introduced size-controllable preparation method of Au NPs with diameter from 2 up to 6 nm by varying the pH of the reaction system^41^, which procedure was recently modified to produce nanoparticles over larger size range^42^. Ohyama *et al*. prepared sulfur-based ligand protected Au NPs in the same size range as Brinas. The size of Au NPs was in this case tuned by adjusting the thiol/HAuCl_4_ ratio^43^. Song *et al*. published the synthesis of Au NPs with a diameter between 1 and 6 nm, where the size control was achieved by changing the fraction of CHCl_3_ in mixture with benzene^44^. Jun *et al*. used milli- and micro-fluidic flow system for the preparation of Au NPs with sizes ranging from 7 to 25 nm. Ascorbic acid was used as reducing agent in this procedure and particle size was tuned by modifying the ascorbic acid *vs*. gold salt concentration ratio and the flow rate of the solutions^45^. Akamatsu *et al*. synthesized nanocomposite microgels with size controlled from 10 up to 30 nm using dimethylamineborane as reducing agent. In this case, the size of prepared Au NPs was controlled by varying the reduction rate^46^. Two step seeded growth method which does not involve application of any surface modifier (except of citrate) during synthetic procedure enabling preparation of size managed gold particles in the size range from 15 up to 300 nm was reported recently^47^. However, all the above mentioned procedures for preparation of Au NPs are rather complicated and do not enable the preparation of larger amounts of nanoparticles necessary for application of nanoparticles in catalysis beyond the laboratory scale.

This work presents a simple and reproducible procedure for one-step preparation of Au NPs that provides a straightforward control of the size of the produced nanoparticles in a size range from 6 up to 22 nm, where the final size of the Au NPs is controlled by varying the concentration of nonionic surfactant Tween 80 (polyethylene glycol sorbitan monooleate) in the reaction mixture. This method of size control allows the realization of a size dependence study on catalytic activity of Au NPs and evaluation of reaction mechanism of the used model reaction. The synthesis procedure is carried out in open vessels, which points to the possibility of large scales essential for application in real practice.

## Results and Discussion

### Synthesis of various-sized Au NPs

**Figure 1:**
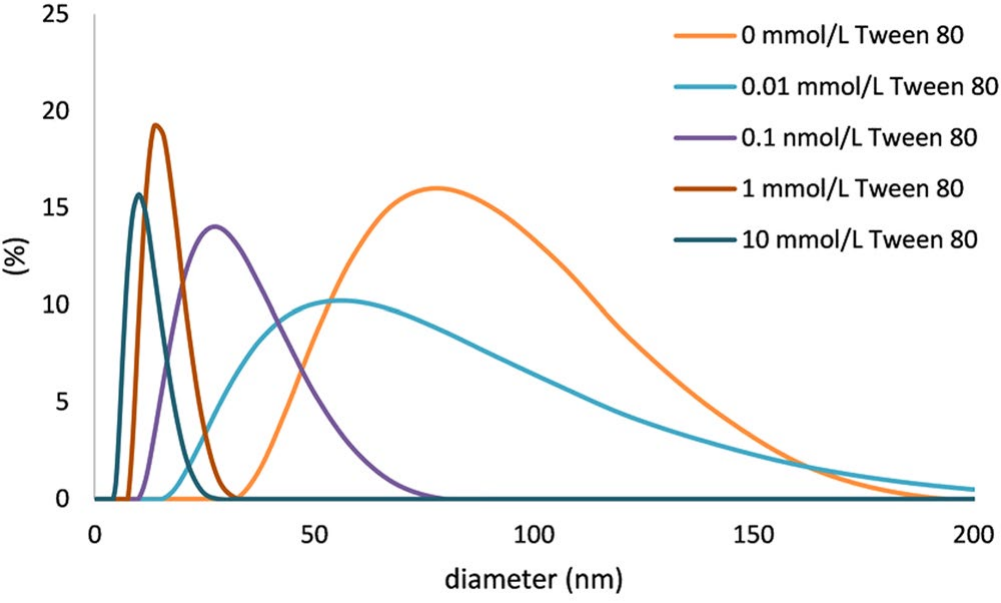
Size distribution of Au NPs obtained by DLS (weighted by intensity) at various concentrations of Tween 80 in the reaction mixture.

Application of variable concentrations of nonionic surfactant Tween 80 in the reaction mixture led into significant changes in the size distribution of the resulting gold nanoparticle dispersions. Figure 1 shows the size distribution (obtained by DLS) of synthesized Au NPs as a function of Tween 80 concentration.

**Figure 2:**
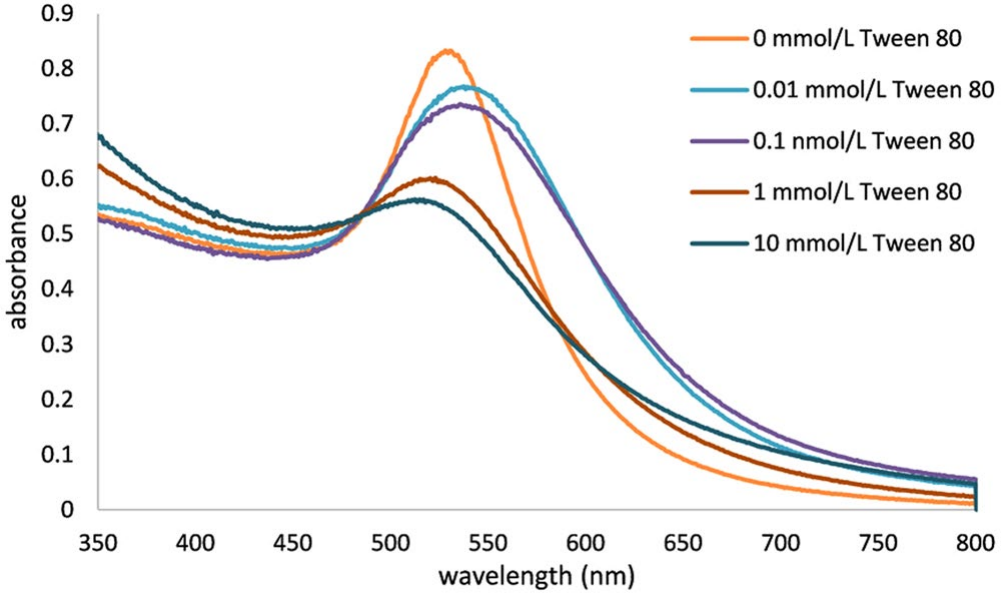
UV-Vis spectra of colloid dispersions containing gold nanoparticles prepared at various concentrations of Tween 80 in the reaction mixture.

**Figure 3:**
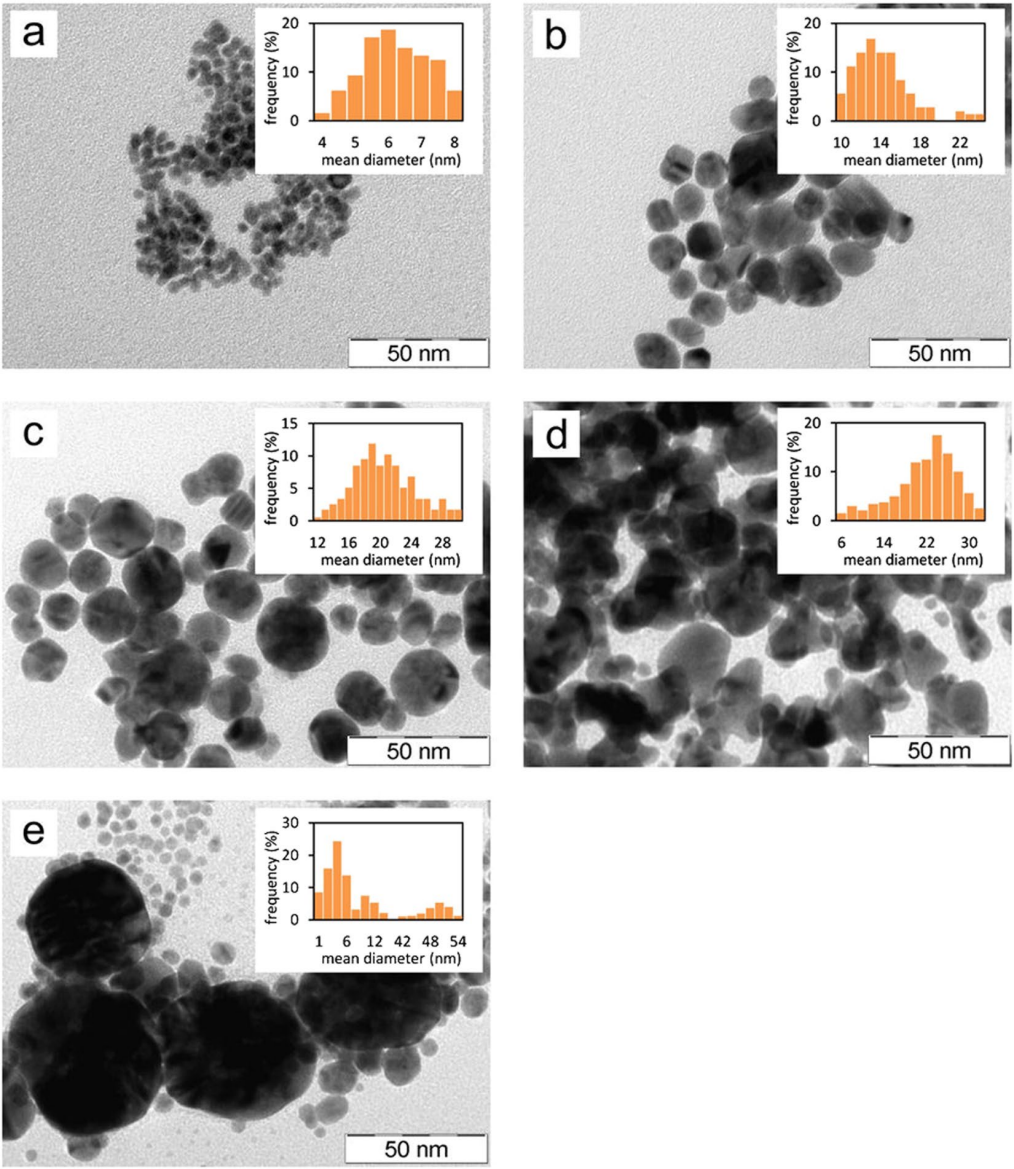
TEM images and particle size distribution histograms of gold nanoparticles prepared via reduction of tetrachloroauric acid by maltose at alkali pH at various concentrations of Tween 80: 10 mmol/L (**a**), 1 mmol/L (**b**), 0.1 mmol/L (**c**), 0.01 mmol/L (**d**), and without addition of Tween 80 (**e**).

According to DLS measurements, increasing concentration of the Tween 80 in the reaction mixture in the range from 0.1 up to 10 mmol/L results in formation of Au NPs with decreasing average diameter from around 80 nm to 10 nm. The observed size differences between samples prepared with various concentration of Tween 80 were qualitatively confirmed by collecting UV-Vis spectra (Fig. 2). Except the non-modified system, the UV-Vis absorption maximum of Au NPs’ surface plasmon is shifted to the lower wavelengths with increasing concentration of Tween 80. This phenomenon reflects the changes of optical properties of Au NPs caused by decreasing particle diameter. Namely, by assuming spherical gold nanoparticles, with decreasing size of the particles the spectrum undergoes a blue shift, while the extinction coefficient drops as well. This explains the shift of the absorption maximum located at 538 nm for 80 nm nanoparticles to 515 nm for the smallest 10 nm nanoparticles, as well as the drop in absorption with decreasing of the size of the nanoparticles^48,49^. There is an apparent discrepancy with the position of the absorption maximum in the case of non-modified system, which could arise from the multimodal particle size distribution with broader size- and shape particle distributions than found for Tween-treated samples (see Table 1). Also some contribution of Tween 80 to the change of the dielectric constant of surrounding media can cause slight red shift of surface plasmon absorption maxima of Tween modified nanoparticles. Although this effect will be subtle (the diffractive index of Tween 80 is about 1.473 in comparison with value for water 1.330) the observed shift between surface plasmon maxima for unmodified and Tween modified may be influenced also by this phenomenon^50,51^. However, based on the available data, an unambiguous determination of the cause of this anomaly cannot be made. The size and morphology of the prepared Au NPs was further studied by transmission electron microscopy (TEM) and typical images are reported on Fig. 3. As it can be seen, in the case of the Au NP dispersions prepared with 0, 0.01 and 0.1 mmol/L of Tween 80 aggregation of the prepared Au NPs takes place, while particle size distribution decreases with the increasing concentration of Tween 80 as also shown by DLS. Moreover, in the case of experiment conducted without Tween 80 a multimodal distribution of the emerged gold colloid is evident with a dominating fraction of very small (<4 nm) particles (Fig. 3). Consequently, this system exhibits maximum of surface plasmon absorbance at a shorter wavelength compared to dispersions of nanoparticles prepared at low concentrations of Tween 80 (0.01 and 0.1 mmol/L) which do not contain such small nanoparticles.

**Table 1:** Mean diameters, standard deviations, polydispersity indexes of the prepared Au NPs dispersions calculated from TEM image analysis and the observed half-times of reduction of Au(III) to Au(0). (See Supplementary Fig. S1 for plot of the half times as function of Tween 80 concentration).

Tween 80 concentration (mmol/L)	relative (molar) ratio Au: Tween 80	mean diameter (nm)	standard deviation	polydispersity index	reaction half-time (s)
0	—	13.4 (unimodal distribution)	17.7	—	48
		5.1/49.4 (bimodal distribution)	3.48/3.50	0.458/0.005	
		3.5/10.7/49.4 (trimodal distribution)	1.71/1.86/3.50	0.236/0.030/0.005	
0.01	100	21.8	5.91	0.073	128
0.1	10	20.0	4.35	0.047	152
1	1	14.1	2.93	0.043	164
10	0.1	6.2	1.00	0.026	184

Similar observation of size dependency of the surface plasmon resonance wavelength were observed for other metal nanoparticles, e.g. Ag nanoparticles in He matrix^52^.

Based on the histograms presented in Fig. 3, the mean diameter ($\bar{d}$) and corrected sample standard deviations (s) were calculated as follows:

1 $$\bar{d}=\frac{{\sum }_{i=0}^{j}{x}_{i}{h}_{i}}{N}$$

2 $$s=\,\sqrt{\frac{1}{N-1}\sum _{i=0}^{j}{h}_{i}{({x}_{i}-\bar{d})}^{2}}$$

where *x*_*i*_ is i^th^ nanoparticle diameter, *h*_*i*_ is frequency of i^th^ diameter and *N* is sum of *h*_*i*_ ($i=(0,1,\ldots ,j)$. The polydispersity indexes (PdI) of the prepared gold colloids were calculated according to ISO 22412:2017:

3 $$PdI={(\frac{s}{\bar{d}})}^{2}$$

**Figure 4:**
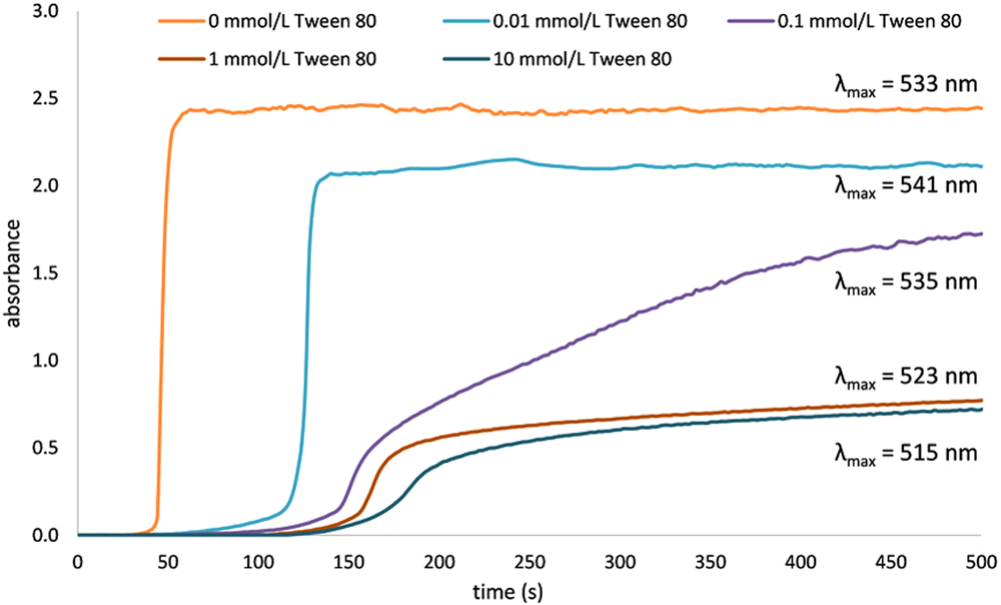
The kinetic curves (based on UV-Vis absorption maxima of emerging gold colloids) of Au(III) reduction by maltose in alkaline pH observed for different concentration of Tween 80 in the reaction mixture.

The results obtained from these calculations are collected in Table 1 along with the Au(III) to Au(0) reduction half-times determined from kinetic curves (Fig. 4). We note that in the case of synthesis without added Tween 80 it was not possible to calculate the corrected standard deviation and the polydispersity index due to a bi/trimodal distribution of the resulting Au NPs. The shortest half-time observed for this sample can be understood due to the absence of the surfactant which may block the active sites of the surface of the formed nuclei and therefore the reaction can proceed without any obstacles. Besides the size control of the Au NPs by the presence of the Tween 80 a significant reduction of polydispersity was observed with decreasing Au NP size: the polydispersity index of Au NPs decreased from 0.073 to 0.026 with increasing concentration of Tween 80 from 0.01 mmol/L to 10 mmol/L.

We assume that the dependence of size and polydispersity of the prepared colloids on the Tween 80 concentration reflects the mode of interaction of Tween 80 with gold species present in the solution, with dominant ion-dipole interaction and strong coordination interaction of gold with hydroxyl groups and oxygens of Tween 80^53,54^. Higher concentrations of Tween 80 enable interaction with a greater fraction of growing gold species which are formed during the reduction process. The surface of these species is partially or fully blocked by adsorbed surfactant molecules. Due to formation of this shielding barrier the catalytic reduction of Au(III) ions on the surface of existing nuclei is slowed down by adsorbed surfactant layer and much slower homogeneous mechanism of formation of new nuclei get the same significance in this case. Also, coalescence of the emerging nuclei and primarily formed very small nanoparticles which is an important step in the growth of the nanoparticles to large crystallites^55^ was suppressed by this surface layer of Tween 80. As a result, the process can lead to the final formation of a greater number of Au NPs with smaller diameter. Blocking the surface by Tween 80 and the slowdown of particle growth is also reflected in the growing delay in the onset of the changes in the UV-Vis spectra with the reaction time (kinetic curves) recorded at the wavelength characteristic for the final particle size at a given concentration of Tween 80 (Fig. 4).

The evolution of the kinetic curves indicate that the formation of Au NPs likely proceeds in several phases^55^. In the first phase, through two interconnected steps, a formation of small Au nuclei (clusters of several atoms of Au) takes place through the reduction of the precursor and these nuclei quickly aggregate to form very small nanoparticles. This first phase of the reaction is reflected in a slow increase (nearly negligible in the non-modified system) of the absorption maxima signal measured at λ_max_, thus the optimum wavelength for the final nanoparticle size. In the second phase of the reaction, the primarily formed nanoparticles are sufficiently large for autocatalytic growth, and therefore they quickly grow almost to their final size, which is reflected by the rapid increase of the absorption signal. In the last reaction phase a slow recrystallization of formed nanoparticles takes place depending on their size. This reaction phase is reflected by very slow and nearly negligible growth of the kinetic curve. According to Fig. 4, in the cases of non-modified system and system modified with the lowest concentration of Tween 80 (0.01 mmol/L), the surface of the forming new nuclei is likely not saturated with the molecules of the surfactant and therefore a fast growth to the final size Au NPs takes place. On the other hand, in the cases of the two highest concentrations of the surfactant (1 mmol/L and 10 mmol/L), the surface of the new nuclei is expected to be highly covered/saturated with Tween 80 which slows down subsequent particle growth in the next reaction steps. The kinetic curve of the system modified by 0.1 mmol/L Tween 80 seems to represent a transition between the above described two extremes.

The difference in the reaction rate between first and consecutive phases becomes smaller with increasing concentration of Tween 80, which is reflected by the changes in slope of the recorded kinetic curves. As principal measurable characteristic of changes in kinetics of the studied reaction could be used the reaction half time which is commonly determined as point of inflexion of the kinetic curve. The obtained dependence of the half time on the concentration of Tween 80 in the reaction system (Table 1, Supplementary Fig. S1) clearly demonstrates the essential influence of Tween 80 on the reaction course. Substantial deceleration of reduction process by the existence of inhibiting layer of used surfactant on the surface emerging Au NPs is then reflected in decrease of their average size. Formation of new nuclei is preferable in the case when surface of existing particles is blocked by the layer of adsorbed molecules of surfactant. This phenomenon results in the observed decrease of final size of the prepared nanoparticles.

### Catalytic activity of various-sized Au NPs

**Figure 5:**
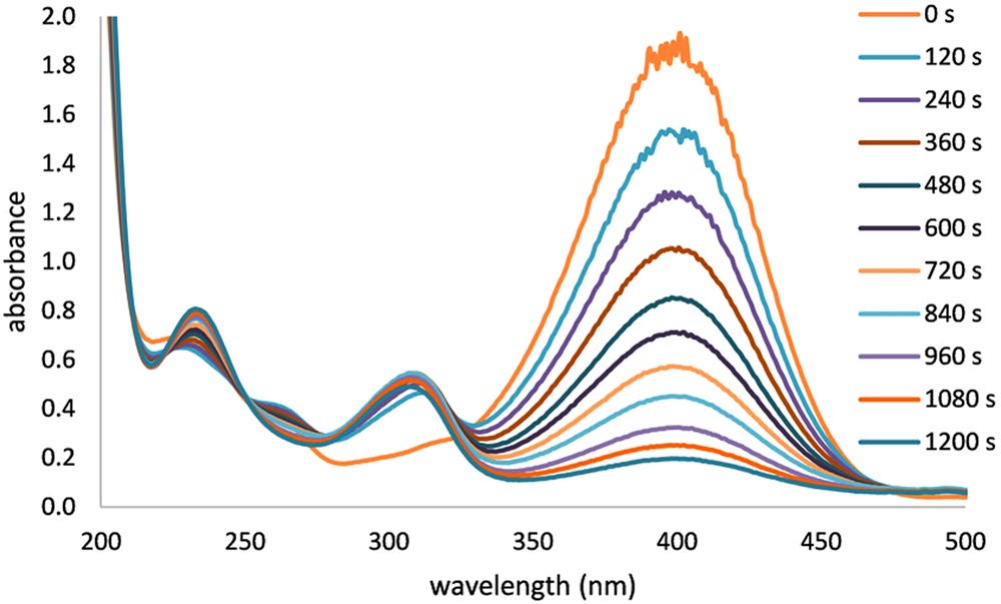
Time-dependent absorption spectra of the solution of 4-nitrophenol during its reduction by sodium borohydride to 4-aminophenol catalysed by Au NPs prepared without Tween 80, recorded in 2 minute intervals. (See Supplementary Fig. S2–S4 online for the spectra recorded in the remaining cases of Au catalytic nanoparticles).

The catalytic activity of the prepared Au NPs was studied using a model reaction based on the reduction of 4-nitrophenol to 4-aminophenol by sodium borohydride. The advantage of this reaction is that its reaction course can be monitored using UV-Vis spectroscopy (Fig. 5 and Supplementary Fig. S2–S5).

Heterogeneous catalytic reactions can be in general described by two kinetic models, namely by Langmuir-Hinshelwood mechanism or Eley-Rideal mechanism, which differ in the adsorption processes of the reactants on surface of catalyst. In the case of the Langmuir-Hinshelwood mechanism, the reaction between two molecules takes place on the catalyst’s surface after adsorption of both reactants onto the active area of catalyst’s surface^56^. In the case of the Eley-Rideal mechanism only one of the reactant molecules is adsorbed on the surface prior to its reaction with the second molecule which collides with the complex without coadsorbing first^57^.

The catalytic reaction according to Langmuir-Hinshelwood model consists of several steps. The first one includes NaBH_4_ reaction on the nanoparticle surface and forming surface-hydrogen species^24,30^. Second step is connected with 4-nitrophenol diffusion from solution and adsorption on the metal surface. Subsequently 4-nitrophenol molecule is reduced by surface-hydrogen species produced by BH_4_^−^ hydrolysis and final reaction step include desorption of reduction products from the surface and their diffusion away from the surface of the catalyst. If diffusion, adsorption and desorption processes are reversible and fast, than the reduction of 4-nitrophenol molecule becomes the rate-determining step^58^. Hence the reaction can be described by Equation 4 (Langmuir-Hinshelwood mechanism), where the catalytic reduction takes place on the surface of gold nanoparticle^57^

4 $$v=-\frac{d{C}_{A}}{dt}=-\frac{d{C}_{B}}{dt}=\frac{k{K}_{A}{K}_{B}{C}_{A}{C}_{B}}{{(1+{K}_{A}{C}_{A}+{K}_{B}{C}_{B})}^{2}}{S}^{2}$$

where *k* is the rate constant of the reaction, *S* is the surface of the catalyst, *K*_*A*_ and *K*_*B*_ represents adsorption coefficients and *C*_*A*_ and *C*_*B*_ are the concentrations of reactants in a reaction mixture (“A” index belongs to 4-nitrophenol, “B” index belongs to NaBH_4_). Because of very low concentration of 4-nitrophenol compared to sodium borohydride (*K*_*A*_*C*_*A*_ ≪ *K*_*B*_*C*_*B*_), it is possible to simplify the kinetic equation (4) to the following form

5 $$v=\frac{k{K}_{A}{K}_{B}{C}_{A}{C}_{B}}{{(1+{K}_{B}{C}_{B})}^{2}}{S}^{2}$$

Substitution of all constants except *C*_*A*_ into an overall constant *k*_*app(L-H)*_ leads to the well-known first order kinetic equation^56^

6 $$v={k}_{app(L-H)}{C}_{A}$$

In the case of the Eley-Rideal mechanism, only one of two reactants is adsorbed onto metal surface which then reacts with the second reactants coming from solution without adsorbing on the surface of the catalyst. Applying the same approach as used above for the Langmuir-Hinshelwood model, the reaction according to Eley-Rideal mechanism can be described by equation (7)^59^

7 $$v=\frac{k{K}_{A}{C}_{A}{C}_{B}}{1+{K}_{A}{C}_{A}}S$$

Substitution of all constants except *C*_*A*_ into an overall constant *k*_*app (E-R)*_ and using presumption *K*_*A*_*C*_*A*_ «1 (very low concentration of reactant A) leads again to the first order kinetic equation

8 $$v={k}_{app(E-R)}{C}_{A}$$

As a result, in both considered cases, the reaction course of 4-nitrophenol reduction can be evaluated as the relative absorbance change (*A*_*t*_*/A*_0_, were *A*_*t*_ and *A*_0_ denotes absorbance at time t and at beginning respectively) at 400 nm (absorption maximum of 4-nitrophenol) with time. The apparent rate constant was determined from the slope of the linear correlation of *ln(A*_*t*_*/A*_0_) with time according to Wunder *et al*.^60^.

**Figure 6:**
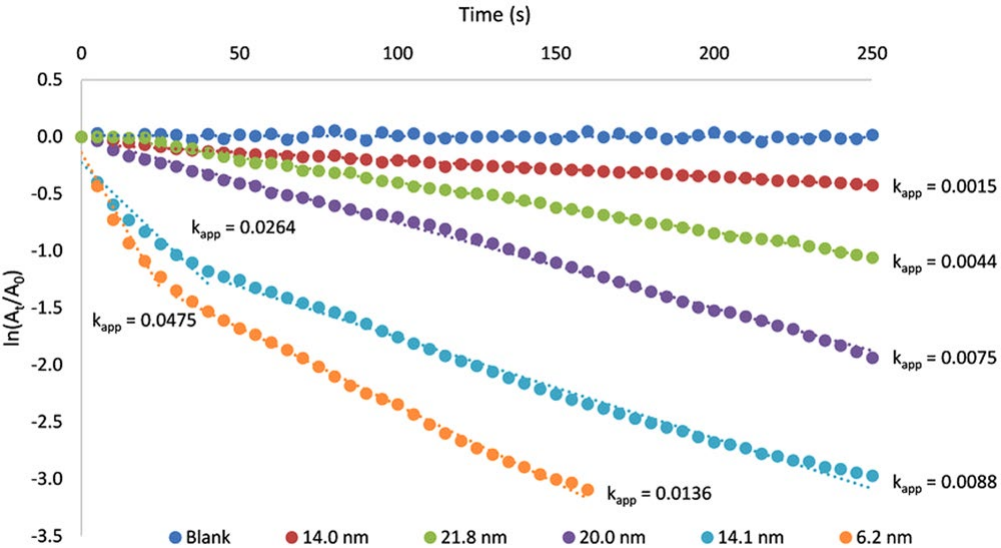
Kinetic curves and apparent rate constants of 4-nitrophenol reduction by sodium borohydride heterogeneously catalysed by various sized gold nanoparticles and without gold nanoparticles (blank).

**Figure 7:**
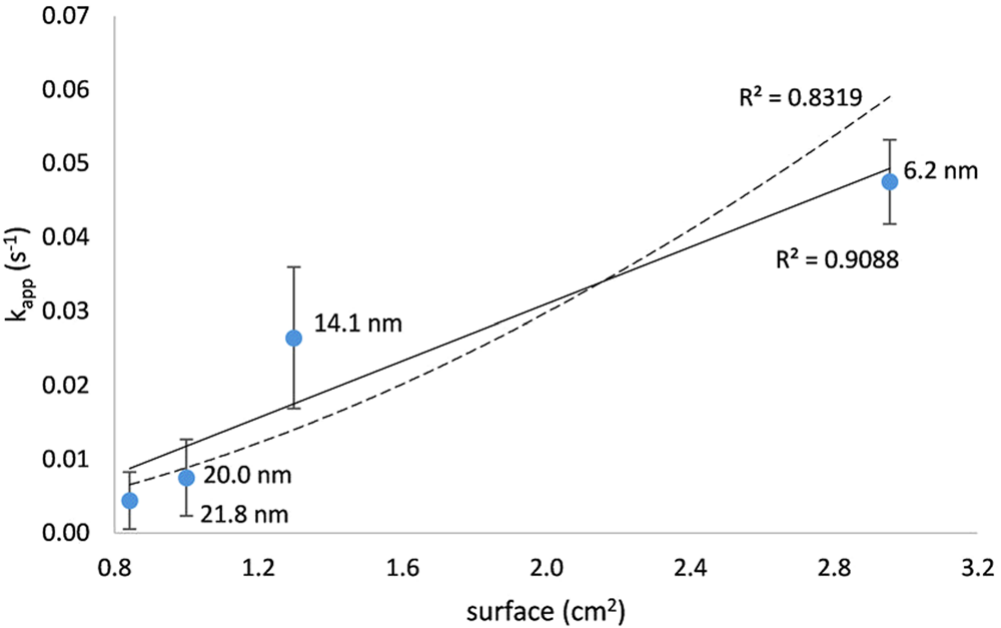
Dependence of the reaction rate of 4-nitrophenol reduction to 4-aminophenol by sodium borohydride catalysed by various sized Au NPs on their total surface at constant amount of gold in the reaction mixture (10 μmol/L). Experimental data are evaluated by linear (full line) and by quadratic function (dashed line).

The obtained kinetic curves (Fig. 6), show the rate of 4-nitrophenol reduction increasing with decreasing Au particle size (see Supplementary Table S1). However, in the case of the two smallest Au NPs (prepared by two highest concentrations of Tween 80, 1 mmol/L and 10 mmol/L), the situation is somewhat complicated. After the initial fast part of the reaction, a slowdown of reaction rate was observed. This effect is most probably caused by saturation of active surface by reaction product 4-aminophenol and its slow desorption. The inhibition of the reaction by the reaction product is a common complication in heterogeneous catalysis, and in the case of studied reaction can be ascribed to the greater affinity of 4-aminophenol to gold surface compared to 4-nitrophenol. The difference in adsorption activity can be assigned to the different mode of adsorption of these two molecules onto gold surface. In the case of 4-aminophenol, the functional group –NH_2_ has character of Lewis base while gold is a typical Lewis acid. The acid-base interaction is therefore responsible for the strong adsorption of 4-aminophenol on gold surface. On the other hand, the 4-nitrophenol lies in flat, parallel orientation on the gold surface, which is typical for van der Waals interaction mediated by the conjugated system of aromatic ring’s π-electrons^61^. The acid-base interaction is generally very strong (energy of this interaction is in the range typical for chemical bond) compared to the weak van der Waals interaction and therefore 4-aminophenol is adsorbed more strongly on the gold surface than 4-nitrophenol. Electrochemical study of the 4-nitrophenol reduction on the gold electrode was performed to support this difference of the reaction components in the adsorption behaviour. The cyclic voltammogram obtained on gold electrode at pH = 10 and scan rate 500 mV/s (Supplementary Fig. S6) confirmed the hypothesis about inhibition of the reaction by the strong adsorption of the reduction products. The reduction peak of 4-nitrophenol observed at −0.820 V (vs. saturated AgCl/Ag reference electrode) during the first scan disappeared at second scan which is a clear evidence of blocking of electrode surface by the reduction product 4-aminophenol^62–64^. Inhibition by slow desorption of the reaction product 4-aminophenol is probably also responsible for the slowdown of the reaction rate after the quick phase observed for smallest catalytic gold nanoparticles (Fig. 6). The turning point on the kinetic curve was observed at the same conversion of the 4-nitrophenol (at about 65%) for both fastest catalytic experiments for which the inhibition is most likely due to the shortest reaction time. In these two cases the reduction rate is so fast that desorption of reaction product 4-aminophenol turns out to be the rate limiting step in the overall reaction mechanism. Therefore, based on the discussed presumption of change in the reaction rate limiting step, the apparent rate constants taken from the beginning quick part of these kinetic curves were used for evaluation of dependence of reaction rate on the total surface of catalyst. Due to the difference between Langmuir-Hinshelwood and Eley-Rideal mechanisms, reflected in the different dependence of reaction rate on the catalyst’s surface, regression of the dependence of the obtained kinetic data on total surface of gold catalyst was conducted. In the case of Langmuir-Hinshelwood model the correlation should be along the quadratic function (Eq. 4) while in the case of Eley-Rideal mechanism the correlation has a linear form (Eq. 7). In the studied cases, kinetic data (apparent rate constants) depend on the total surface of gold catalyst linearly (coefficient of determination 0.9088) as predicted by the Eley-Rideal mechanism. However, if the same data are interpolated by the quadratic function as is true in the case of Langmuir-Hinshelwood model the correlation is significantly worse, as the correlation coefficient is only 0.8319 (Fig. 7). As reported in the literature, for a typical reaction which fulfils this model (e.g. the reduction of methylene blue with NaBH_4_ catalysed by Ag NPs), a correlation of the data with quadratic dependency k_app_ = f(S^2^) is much better (correlation coefficient 0.949)^56^.

The catalytic efficiency of the prepared Au NPs was compared with literature data on the basis of the apparent rate constants normalized to the same concentration of gold in the reaction system (Supplementary Table S2). Attention was paid to studies which were aimed at application of surface modified Au NPs in the same model reaction as was used in this study.

Primarily, the reported results confirm the strong effect of the size of Au NPs on their catalytic activity. The apparent rate constants are steeply growing with decreasing size of nanoparticles due to growth of total surface area at the same concentration of the catalyst in the reaction system. Additionally to this main effect, the catalytic activity is also dependent on the surface modification of the nanoparticles. Modification of the catalyst surface by citrate^40^ has nearly the same effect as in the case of Tween 80 used in this study. Some improvement of the catalytic activity of Au NPs were obtained using azacryptand (uncharged molecule with N heteroatom – Lewis base) as surface modifier^40^ in comparison with citrate or Tween 80. On the other hand, the application of cationic surfactant cetyltrimethylammonium bromide (CTAB; positively charged molecule with N heteroatom – Lewis base) as surface modifier lead to significant decrease in of catalytic activity^61^. This effect is clearly connected with change in surface charge of the catalyst from negative (typical sign of charge of Au NPs in alkaline aqueous media) to positive value due to adsorption of the cationic surfactant.

In summary, the presented study introduces a controlled preparation of Au NPs in the range from 6 up to 22 nm. The size of Au NPs was controlled by varying the concentration of Tween 80, which, controls the size and the polydispersity of resulting Au NPs. Higher concentration of Tween 80 results in interaction with a greater number of growing gold nuclei which are formed during the initial phase of the reduction process. With their surface blocked by Tween 80, the formation of new nuclei is preferred over the further growth of the preformed nuclei, as reflected by slowdown of the reduction rate and in final formation of smaller nanoparticles with increasing concentration of the surfactant used during the synthesis of the Au NPs. Analysis of the kinetic data obtained from catalytic study with the prepared Au NPs reveals that reduction of 4-nitrophenol is going in this case along with the Eley-Rideal mechanism of heterogeneous catalysis.

## Materials and Methods

### Chemicals

Tetrachloroauric acid trihydrate (Merck Millipore, for analysis), D(+)-Maltose monohydrate (Sigma-Aldrich, p.a.), sodium hydroxide (Lach-Ner, p.a.), Tween 80 (Sigma-Aldrich, p.a.), 4-nitrophenol (Sigma-Aldrich, p.a.), sodium borohydride (Sigma-Aldrich, p.a.) were used as received without additional purification. Deionized water (18 MΩ·cm, Millipore) was used to prepare all solutions.

### Synthesis of various-sized Au NPs

The colloidal dispersions of Au NPs were prepared by the reduction of tetrachloroauric acid with saccharide maltose at the laboratory temperature. The final size of resulting Au NPs was controlled by varying the amount of nonionic surfactant Tween 80 present in the solution. In the case of the solution free of Tween 80, 5 ml of the 5 mmol/L tetrachloroauric solution was diluted with 10 ml of deionized water. In the case of solutions with added Tween 80, 25 mmol/L of Tween 80 solution was first added to the 5 mmol/L tetrachloroauric solution, then the solution was topped up to 15 ml with deionized water. The final concentration of Tween 80 in the reaction system ranged between 0.01 mmol/L and 10 mmol/L. The solutions were vigorously stirred using a magnetic stirrer. The reduction was started by the addition of 10 ml of reducing solution containing maltose and sodium hydroxide both at concentration of 25 mmol/L. The formation of the nanoparticles was accompanied with a color change from light yellow to dark red or violet depending on the resulting Au NPs size. The size distribution of the prepared Au NPs was determined by DLS and UV-Vis spectroscopy after completing of the reaction (30 minutes). The UV-Vis spectra were recorded using samples diluted five times due to high absorbance of the produced dispersions. Complementary characterization of the particles was performed by transmission electron microscopy (TEM). The samples for catalytic testing were left in an open beaker for 24 hours to remove all unreacted sodium borohydride.

### Characterization techniques

The size of the prepared Au NPs was determined using dynamic light scattering (DLS, Zetasizer Nano ZS, Malvern Instruments, UK) and verified by transmission electron microscopy (TEM, JEM 2100, Jeol Ltd., Japan). Samples for TEM were prepared simple by sedimentation of Au NPs from a drop of dispersion on the microscopic copper TEM grid with carbon layer. Excess of the dispersion was removed by filtration paper and the grids with the Au NPs were dried free on the air (covered by the Petri dish) at laboratory temperature. The nanoscopic character of the prepared gold particles was further confirmed by UV-Vis spectroscopy (Specord S600, Analytic Jena AG, Germany), which was also used for the determination of the concentration of 4-nitrophenol in the catalytic experiments.

### Catalytic activity measurements

The catalytic activity of the synthesized Au NPs was tested using the model reaction of 4-nitrophenol (4-NP) reduction to 4-aminophenol (4-AP) by sodium borohydride at pH 10 (adjusted by 1 mol/L NaOH aqueous solution). The consumption of 4-nitrophenol was monitored by the decrease of its absorption peak (located at 400 nm) with time. For the purposes of the kinetic experiments, 2 ml of the 1.5 mmol/L solution of 4-nitrophenol was mixed with 0.47 ml of distilled water and 0.03 ml of the prepared gold colloids at Au concentration of 1 mmol/L in a quartz cuvette. The solution was being mixed by shaking the cuvette. The reaction was initiated by rapid injection of 0.5 ml of a 10 mmol/L solution of sodium borohydride, and the cuvette was quickly placed into the UV-Vis spectrometer for the collection of spectra as a function of time. The kinetic experiments were repeated three times for confirmation of the reproducibility of the catalytic activity of the tested Au NPs.

### Calculation of the surface of the nanocatalyst

For the purpose of catalytic activity evaluation of the total surface area per sample of the prepared Au NPs was calculated based on their particle size determined by TEM and the total amount of gold in the reaction mixture. The morphology of the prepared Au NPs was approximated for this calculation by a sphere with diameter equal to the average diameter of the particles in dispersion determined from the TEM images.

## Electronic supplementary material

Supplementary Information

## References

[1] Sattarahmady N, Movahedpour A, Heli H, Hatam GR (2016). Gold nanoparticles-based biosensing of Leishmania major kDNA genome: Visual and spectrophotometric detections. Sensors Actuators B Chem..

[2] Kacanovska A, Rong Z, Schmidt M, Russell PSJ, Vadgama P (2010). Bio-sensing using recessed gold-filled capillary amperometric electrodes. Anal. Bioanal. Chem..

[3] Liu Y (2014). Development of gold nanoparticle-sheathed glass capillary nanoelectrodes for sensitive detection of cerebral dopamine. Biosens. Bioelectron..

[4] Chen Q (2013). Targeted CT/MR dual mode imaging of tumors using multifunctional dendrimer-entrapped gold nanoparticles. Biomaterials.

[5] Bian P, Zhou J, Liu Y, Ma Z (2013). One-step fabrication of intense red fluorescent gold nanoclusters and their application in cancer cell imaging. Nanoscale.

[6] Cortes Vega FD (2017). Gold nanoparticle SERS substrates sustainable at extremely high temperatures. J. Mater. Chem. C.

[7] Li Y, Qi X, Lei C, Yue Q, Zhang S (2014). Simultaneous SERS detection and imaging of two biomarkers on the cancer cell surface by self-assembly of branched DNA-gold nanoaggregates. Chem. Commun. (Camb)..

[8] Du Y (2014). Enhanced light–matter interaction of graphene–gold nanoparticle hybrid films for high-performance SERS detection. J. Mater. Chem. C.

[9] Kong D (2016). A gold nanoparticle-based semi-quantitative and quantitative ultrasensitive paper sensor for the detection of twenty mycotoxins. Nanoscale.

[10] Campbell, C. T. The Active Site in Nanoparticle Gold Catalysis. *Science* (*80-)*. **306** (2004). 10.1126/science.1104246 15472065

[11] Hu W (2014). Sensitive detection of multiple mycotoxins by SPRi with gold nanoparticles as signal amplification tags. J. Colloid Interface Sci..

[12] Guo Y (2014). Label-Free Colorimetric Detection of Cadmium Ions in Rice Samples Using Gold Nanoparticles. Anal. Chem..

[13] Marie-Christine, D. & Astruc, D. Gold Nanoparticles: Assembly, Supramolecular Chemistry, Quantum-Size-Related Properties, and Applications toward Biology, Catalysis, and Nanotechnology, 10.1021/CR030698+ (2003). 10.1021/cr030698+ 14719978

[14] Corma A (2008). Supported gold nanoparticles as catalysts for organic reactions. Chem. Soc. Rev..

[15] Méndez-Cruz M, Ramírez-Solís J, Zanella R (2011). CO oxidation on gold nanoparticles supported over titanium oxide nanotubes. Catal. Today.

[16] Haruta M (1997). Size- and support-dependency in the catalysis of gold. Catal. Today.

[17] Valden M (1998). Onset of Catalytic Activity of Gold Clusters on Titania with the Appearance of Nonmetallic Properties. Science (80-.)..

[18] Ide MS, Davis RJ (2014). The important role of hydroxyl on oxidation catalysis by gold nanoparticles. Acc. Chem. Res..

[19] Liu W, Yang X, Xie L (2007). Size-controlled gold nanocolloids on polymer microsphere-stabilizer via interaction between functional groups and gold nanocolloids. J. Colloid Interface Sci..

[20] Huang X, Liao X, Shi B (2011). Synthesis of highly active and reusable supported gold nanoparticles and their catalytic applications to 4-nitrophenol reduction. Green Chem..

[21] Rahman Zur (2014). Preparation and characterization of magnetic gold shells using different sizes of gold nanoseeds and their corresponding effects on catalysis. RSC Adv..

[22] Sinha AK, Seelan S, Tsubota S, Haruta M (2004). Catalysis by Gold Nanoparticles: Epoxidation of Propene. Top. Catal..

[23] Lee S (2009). Selective propene epoxidation on immobilized Au6-10 clusters: The effect of hydrogen and water on activity and selectivity. Angew. Chemie - Int. Ed..

[24] Takale BS, Bao M, Yamamoto Y (2014). Gold nanoparticle (AuNPs) and gold nanopore (AuNPore) catalysts in organic synthesis. Org. Biomol. Chem..

[25] Taketoshi A, Haruta M (2014). Size- and Structure-specificity in Catalysis by Gold Clusters. Chem. Lett..

[26] Tsunoyama H, Sakurai H, Tsukuda T (2006). Size effect on the catalysis of gold clusters dispersed in water for aerobic oxidation of alcohol. Chem. Phys. Lett..

[27] Jawale DV (2014). Size effect of gold nanoparticles supported on carbon nanotube as catalysts in selected organic reactions. Tetrahedron.

[28] Yang M-Q, Pan X, Zhang N, Xu Y-J (2013). A facile one-step way to anchor noble metal (Au, Ag, Pd) nanoparticles on a reduced graphene oxide mat with catalytic activity for selective reduction of nitroaromatic compounds. CrystEngComm.

[29] Aromal SA, Babu KVD, Philip D (2012). Characterization and catalytic activity of gold nanoparticles synthesized using ayurvedic arishtams. Spectrochim. Acta. A. Mol. Biomol. Spectrosc..

[30] Wunder S, Polzer F, Lu Y, Mei Y, Ballauff M (2010). Kinetic Analysis of Catalytic Reduction of 4-Nitrophenol by Metallic Nanoparticles Immobilized in Spherical Polyelectrolyte Brushes. J. Phys. Chem. C.

[31] Lin S-Y (2010). The protease-mediated nucleus shuttles of subnanometer gold quantum dots for real-time monitoring of apoptotic cell death. J. Am. Chem. Soc..

[32] Lin C, Tao K, Hua D, Ma Z, Zhou S (2013). Size effect of gold nanoparticles in catalytic reduction of p-nitrophenol with NaBH4. Molecules.

[33] Wang Y-G, Mei D, Glezakou V-A, Li J, Rousseau R (2015). Dynamic formation of single-atom catalytic active sites on ceria-supported gold nanoparticles. Nat. Commun..

[34] Herzing AA, Kiely CJ, Carley AF, Landon P, Hutchings GJ (2008). Identification of Active Gold Nanoclusters on Iron Oxide Supports for CO Oxidation. Science (80-.)..

[35] Boronat M, Leyva-Pérez A, Corma A (2014). Theoretical and Experimental Insights into the Origin of the Catalytic Activity of Subnanometric Gold Clusters: Attempts to Predict Reactivity with Clusters and Nanoparticles of Gold. Acc. Chem. Res..

[36] Turkevich J (1985). Colloidal gold. Part I. Gold Bull..

[37] Martin MN, Basham JI, Chando P, Eah S-K (2010). Charged Gold Nanoparticles in Non-Polar Solvents: 10-min Synthesis and 2D Self-Assembly. Langmuir.

[38] Chen H, Wang YY, Dong S, Wang E (2006). One-step preparation and characterization of PDDA-protected gold nanoparticles. Polymer (Guildf)..

[39] Li M-D, Cheng T-L, Tseng W-L (2009). Nonionic surfactant-capped gold nanoparticles for selective enrichment of aminothiols prior to CE with UV absorption detection. Electrophoresis.

[40] Lee KY, Hwang J, Lee YW, Kim J, Han SW (2007). One-step synthesis of gold nanoparticles using azacryptand and their applications in SERS and catalysis. J. Colloid Interface Sci..

[41] Briñas RP, Hu M, Qian L, Lymar ES, Hainfeld JF (2008). Gold nanoparticle size controlled by polymeric Au(I) thiolate precursor size. J. Am. Chem. Soc..

[42] Piella J, Bastús NG, Puntes V (2016). Size-Controlled Synthesis of Sub-10-nanometer Citrate-Stabilized Gold Nanoparticles and Related Optical Properties. Chem. Mater..

[43] Ohyama J, Hitomi Y, Higuchi Y, Tanaka T (2009). Size Controlled Synthesis of Gold Nanoparticles by Porphyrin with Four Sulfur Atoms. Top. Catal..

[44] Song J, Kim D, Lee D (2011). Size control in the synthesis of 1-6 nm gold nanoparticles via solvent-controlled nucleation. Langmuir.

[45] Jun H (2012). Understanding of the size control of biocompatible gold nanoparticles in millifluidic channels. Langmuir.

[46] Akamatsu K (2010). Synthesis of pH-responsive nanocomposite microgels with size-controlled gold nanoparticles from ion-doped, lightly cross-linked poly(vinylpyridine). Langmuir.

[47] Ziegler C, Eychmüller A (2011). Seeded Growth Synthesis of Uniform Gold Nanoparticles with Diameters of 15−300 nm. J. Phys. Chem. C.

[48] Rance GA, Marsh DH, Khlobystov AN (2008). Extinction coefficient analysis of small alkanethiolate-stabilised gold nanoparticles. Chem. Phys. Lett..

[49] Zuber A (2016). Detection of gold nanoparticles with different sizes using absorption and fluorescence based method. Sensors Actuators B Chem..

[50] Jensen TR (1999). Nanosphere Lithography: Effect of the External Dielectric Medium on the Surface Plasmon Resonance Spectrum of a Periodic Array of Silver Nanoparticles. J. Phys. Chem. B.

[51] Kelly KL (2003). The Optical Properties of Metal Nanoparticles: The Influence of Size, Shape, and Dielectric Environment. J. Phys. Chem. B.

[52] Loginov E (2011). Photoabsorption of AgN(N∼6–6000) Nanoclusters Formed in Helium Droplets: Transition from Compact to Multicenter Aggregation. Phys. Rev. Lett..

[53] Jia H (2011). Siloxane surfactant induced self-assembly of gold nanoparticles and their application to SERS. CrystEngComm.

[54] Zhou H, Zheng L, Jia H (2014). Facile control of the self-assembly of gold nanoparticles by changing the capping agent structures. Colloids Surfaces A Physicochem. Eng. Asp..

[55] Polte J (2010). Mechanism of Gold Nanoparticle Formation in the Classical Citrate Synthesis Method Derived from Coupled *In Situ* XANES and SAXS Evaluation. J. Am. Chem. Soc..

[56] Panacek A (2014). Polyacrylate-Assisted Size Control of Silver Nanoparticles and Their Catalytic Activity. Chem. Mater..

[57] Atkins, P. & Paula, J. De. *Atkins‘ Physical Chemistry*. (WH Freeman, 2010).

[58] Hervés P (2012). Catalysis by metallic nanoparticles in aqueous solution: model reactions. Chem. Soc. Rev..

[59] Logan, S. R. *Fundamentals of Chemical Kinetics*. (Longman, 1996).

[60] Wunder S, Lu Y, Albrecht M, Ballauff M (2011). Catalytic Activity of Faceted Gold Nanoparticles Studied by a Model Reaction: Evidence for Substrate-Induced Surface Restructuring. ACS Catal..

[61] Fenger R, Fertitta E, Kirmse H, Thünemann AF, Rademann K (2012). Size dependent catalysis with CTAB-stabilized gold nanoparticles. Phys. Chem. Chem. Phys..

[62] Ikhsan NI, Rameshkumar P, Huang NM (2016). Controlled synthesis of reduced graphene oxide supported silver nanoparticles for selective and sensitive electrochemical detection of 4-nitrophenol. Electrochim. Acta.

[63] Luo L, Zou X, Ding Y, Wu Q (2008). sheng. Derivative voltammetric direct simultaneous determination of nitrophenol isomers at a carbon nanotube modified electrode. Sensors Actuators, B Chem..

[64] Liu Z (2009). Electrochemical sensor for detection of p-nitrophenol based on nanoporous gold. Electrochem. commun..

