# Supplementary material for: Simple size-controlled synthesis of Au nanoparticles and their size-dependent catalytic activity
Source: Sci Rep. 2018 Mar 15;8:4589. doi: 10.1038/s41598-018-22976-5 (PMC5854582; doi:10.1038/s41598-018-22976-5)
Supplement: Supplementary file 1 — Supplementary Information [file 41598_2018_22976_MOESM1_ESM.docx]

Simple size-controlled synthesis of Au nanoparticles and their size-dependent catalytic activity

Petr Suchomel^1^, Libor Kvitek^1*^, Robert Prucek^1^, Ales Panacek^1^, Avik Halder^2^, Stefan Vajda^2,3^ and Radek Zboril^1^

^1^ Regional Centre of Advanced Technologies and Materials, Department of Physical Chemistry, Faculty of Science, Palacky University Olomouc, Slechtitelu 27, 783 71 Olomouc, Czech Republic.

^2^ Materials Science Division, Argonne National Laboratory, 9600 South Cass Avenue, Lemont, Illinois 60439, USA

^3^ Institute for Molecular Engineering, The University of Chicago, 5640 South Ellis Avenue, Chicago. Illinois 60637, USA

^*^ Corresponding author: Libor Kvitek, libor.kvitek@upol.cz

**Figure S1**: Evaluation of reaction half-time of gold nanoparticles preparation in the presence of various concentrations of Tween 80.

Figure S2: Absorption spectra of the solution of 4-nitrophenol during its reduction by sodium borohydride to 4-aminophenol as a function of time catalyzed by Au NPs with mean size of 21.8 nm.

Figure S3: Absorption spectra of the solution of 4-nitrophenol during its reduction by sodium borohydride to 4-aminophenol as a function of time catalyzed by Au NPs with mean size of 20.0 nm.

Figure S4: Absorption spectra of the solution of 4-nitrophenol during its reduction by sodium borohydride to 4-aminophenol as a function of time catalyzed by Au NPs with mean size of 14.1 nm.

Figure S5: Absorption spectra of the solution of 4-nitrophenol during its reduction by sodium borohydride to 4-aminophenol as a function of time catalyzed by Au NPs with mean size of 6.2 nm.

Figure S6: The cyclic voltamogram of 4-nitrophenol obtained on gold electrode at pH=10 and scan rate 500 mV/s. In the three-electrode system saturated AgCl/Ag electrode was used as reference electrode and Pt strip as auxiliary electrode (1. scan: black line; 2. scan: red line).

Table S1: Apparent rate constants (initial - * and following # part of kinetic curve) of 4-nitrophenol reduction in the presence of various sized Au NPs.

| Au NPs diameter  (nm) | total surface  (cm^2^) | k_app_  (s^-1^) |
| --- | --- | --- |
| 21.8 | 0.84 | 0.0044 |
| 20.0 | 0.92 | 0.0075 |
| 14.1 | 1.30 | 0.0264* / 0.0088^#^ |
| 6.2 | 2.96 | 0.0475* / 0.0136^#^ |

**Table S2:** Comparison of the apparent rate constants obtained for model reaction of 4-nitrophenol reduction using borohydride catalysed by various gold nanoparticles.

| Size of Au NPs [nm] | k_app_ [s^-1^] | Au content [μmol/L] | k’_app_  [L∙s^-1^μmol Au^-1^] | Surface modification | Ref. |
| --- | --- | --- | --- | --- | --- |
| 21.8 | 0.0044 | 10 | 0.0004 | Tween 80 | this study |
| 20.0 | 0.0075 | 10 | 0.0008 | Tween 80 | this study |
| 20 | 0.01 | 3.07 | 0.0014 | azacryptant | ^1^ |
| 20 | 0.002 | 3.92 | 0.0005 | citrate | ^1^ |
| 14.1 | 0.0264 | 10 | 0.0026 | Tween 80 | this study |
| 15 | 0.0108 | 4.95 | 0.0022 | Immobilized onto Fe_3_O_4_@SiO_2_ | ^2^ |
| 12.9 | 0.0061 | 4.1 | 0.0015 | CTAB | ^3^ |
| 6.2 | 0.0475 | 10 | 0.0048 | Tween 80 | this study |
| 3 | 0.0012 | 4.03 | 0.0003 | CTAB | ^3^ |

**References**

1. Lee, K. Y., Hwang, J., Lee, Y. W., Kim, J. & Han, S. W. One-step synthesis of gold nanoparticles using azacryptand and their applications in SERS and catalysis. *J. Colloid Interface Sci.* **316,** 476–81 (2007).

2. Rahman, Z. ur *et al.* Preparation and characterization of magnetic gold shells using different sizes of gold nanoseeds and their corresponding effects on catalysis. *RSC Adv.* **4,** 5012 (2014).

3. Fenger, R., Fertitta, E., Kirmse, H., Thünemann, A. F. & Rademann, K. Size dependent catalysis with CTAB-stabilized gold nanoparticles. *Phys. Chem. Chem. Phys.* **14,** 9343–9 (2012).
